# Cysteinyl Leukotriene and Systemic Inflammatory Levels in Preeclampsia

**DOI:** 10.7759/cureus.37764

**Published:** 2023-04-18

**Authors:** Gokhan Guzeltas, Mujde can Ibanoglu, Yaprak Engin-Üstün

**Affiliations:** 1 Obstetrics, University of Health Sciences Ankara City Hospital, Ankara, TUR; 2 Obstetrics, Etlik Zubeyde Hanım EAH, Ankara, TUR; 3 Obstetrics and Gynecology, University of Health Sciences Etlik Zubeyde Hanim Women’s Health Training and Research Hospital, Ankara, TUR; 4 Obstetrics and Gynecology, Zekai Tahir Burak Womens Health Research and Education Hospital, Ankara, TUR

**Keywords:** c-reactive protein, cysteinyl leukotriene, systematic inflammation, severe preeclampsia, preeclampsia

## Abstract

Background

In this study, we aimed to demonstrate the efficacy of cysteinyl leukotriene levels, which play a role in inflammation, in predicting the severity of preeclampsia (PE) and to determine whether this marker can be used as a screening tool.

Methods

In this cross-sectional analytic study, we classified pregnant women who were normotensive (control) or PE or severe PE (SPE) between March 2019 and July 2019. Singleton pregnant 60 women who met the following criteria for the diagnosis of PE were included in the study group. We identified 30 patients with PE and 30 patients with SPE. Normotensive pregnant women (n=30) who met this criterion were included as a control group by randomly selecting them on odd days of the week.

Results

All pregnant women who participated in the study had a singleton pregnancy, and maternal age ranged from 18 to 40 years, with a mean age of 28.77±6.37 years. The mean gestational week of the group was 35.54±3.247 weeks. Gestational age was higher in women in the control group (p=0.018), shock index was higher in women in the control group (p < 0.001), and body mass index (BMI) value was lower in this group than in the other groups (p=0.002). The values of mean arterial pressure (MAP) were found to have a strong correlation with shock index value and a weak and negative correlation with gestational week and platelet/lymphocyte ratio (p < 0.05). The mean cysteinyl leukotriene levels of 206.15 pg/mL for the control group, 273.2 pg/mL for PE, and 211.85 pg/mL for SPE were calculated. However, no statistically significant difference was found between the groups (p=0.707).

Conclusion

We found that cysteinyl leukotrienes were not clinically important in assessing the risk for developing PE and predicting SPE. Alanine aminotransferase, white blood cell, lymphocyte, C-reactive protein, platelet/lymphocyte ratio, and shock index were positively correlated with the value of MAP.

## Introduction

Preeclampsia (PE) is a multisystemic progressive disease with a prevalence of 5-8%; it is associated with morbidity and mortality and characterized by hypertension and significant end-organ damage with or without proteinuria in the last half of pregnancy or in the postpartum period [1,2]. Screening pregnant women for risk factors for PE is critical for monitoring pregnancy and planning delivery. The use of laboratory tests and risk factor imaging to predict a woman's risk of developing PE is currently under investigation, as risk factors can predict the development of PE in only 30% of women [3]. However, an established marker for PE has not yet been defined [4]. A systematic review of studies evaluating clinically available tests indicates that the tests are not sensitive or specific enough to screen the general obstetric population and points to the overall poor methodological quality of the available studies [5].

Cysteinyl leukotrienes (CysLTs) are a product of the metabolism of arachidonic acid from membrane phospholipids [6]. They are mediators involved in inflammation and are mainly synthesized by many inflammatory cells such as mast cells, basophils, eosinophils, and macrophages [7]. Recently, neutrophils were found to be activated in the placental bed in PE [8]. These neutrophils contain substances such as elastase and protease that cause vascular damage [9]. In addition, leukotrienes, which are products of arachidonic acid metabolism in maternal leukocytes, can cause increased vascular permeability, vasoconstriction, activation of neutrophils, and inflammation [9]. In PE, the levels of leukotriene produced by neutrophils are increased. Neutrophils, macrophages, and T lymphocytes contribute to vascular injury [10]. CysLTs, a product of the leukotriene family, are considered potent inflammatory mediators that trigger and promote a variety of biological responses both directly and indirectly [11].

Our aim in this study is to demonstrate the efficacy of CysLT levels, which play a role in inflammation, in predicting the severity of PE and to determine whether this marker can be used as a screening tool.

## Materials and methods

In this cross-sectional analytical study, we examined and classified pregnant women who were admitted to the delivery room or perinatology outpatient clinic of Zekai Tahir Burak Women's Health Training and Research Hospital, College of Health Sciences between March 2019 and July 2019 and were normotensive (control) or PE or severe PE (SPE) ( Ethics Committee of School of Health Sciences Zekai Tahir Burak Women's Health Care, Training and Research Hospital on 03/19/2019 # 39/2019).

The study group included 60 single pregnant women diagnosed with preeclampsia. The diagnosis PE was made in accordance with the 2019 American College of Obstetricians and Gynecologists guidelines [12]. Normotensive pregnant women (n=30) who met this criterion were randomly assigned to even days of the week and served as a control group. The characteristics of 90 patients (PE n=30; SPE n=30; normotensive n=30) who became pregnant between 18 and 40 years of age and had a single pregnancy were obtained from the database and medical records. Exclusion criteria were multiple pregnancies, systemic or chronic diseases (hypertension, diabetes, liver disease, heart disease, rheumatic disease, kidney disease, etc.), peripartum complications such as rupture of the membranes, chorioamnionitis, or fetal anomalies, and corticosteroid or active drug use.

Data on age, gravidity, body mass index, and blood pressure (systolic, diastolic, and mean arterial blood pressure (2*diastolic blood pressure (DBP) + systolic blood pressure (SBP))/3)) were collected.

Patients' complete blood counts and biochemical parameters were obtained from records on the day of hospitalization. Complete blood count parameters were obtained using the ADVIA 2120i (Siemens Healthcare, Erlangen, Germany), and biochemical parameters were determined using Beckman Coulter AU680 and AU480 devices. Neutrophil/lymphocyte ratio (NLR) was calculated by dividing neutrophil count by lymphocyte count, and platelet/lymphocyte ratio (PLR) was also calculated by dividing platelet count by lymphocyte count.

Samples for determination of CysLT level were brought to the laboratory within 30 minutes and centrifuged at 2,000 rpm for 20 minutes in the NF800 centrifugation system of Nuve (Nüve Sanayi Malzemeleri İmalat ve Ticaret A.Ş, Ankara, Turkey). Serum samples were filled into Eppendorf tubes and stored at -80˚C until the day of analysis. The collected serum samples were stored in the laboratory of Macrocel-Special Seyrantepe European Dialysis Center at room temperature for about half an hour using Human CysLTs enzyme-linked immunosorbent assay (ELISA) Kits (catalog number SG-01038, SinoGeneclon Biotech Co Ltd, Hangzhou, China). After washing with Microplate Washer RT 2600C (China), the sample was evaluated with Rayto Microplate Reader RT 2100C (Rayto, Shenzhen, China) using 450 wavelength immunoassay method in the laboratory of Macrocel-Special Seyrantepe European Dialysis Center. The lowest concentration that could be reliably detected with the kits used was 10 pg/mL and was within the reference range of 78 pg/mL to 2,500 pg/mL. Values were measured in accordance with the manufacturer's instructions. Microsta, a statistical computer program, was used to calculate the CysLT levels in the samples based on the results of the ELISA studies. Optical density (OD) values of standards with known concentrations were used. The values were subjected to regression correlation analysis, and the concentrations of the samples were calculated.

Statistical Package for the Social Sciences (SPSS) Version 22 (SPSS Inc., Armonk, NY) was used for statistical analysis. The distribution of parameters was assessed using the Shapira-Wilk normality tests. Data were expressed as mean and standard deviation. For normally distributed data, the independent-samples t-test was used, and for nonnormally distributed variables, the Mann-Whitney U test was used. ROC (receiver operating characteristic) curve analyses were performed to determine the appropriate cut-off point for the independent markers and to calculate sensitivity and specificity values. A statistically significant p-value was assessed as less than 0.05.

The power analysis for sample size calculation was based on the previous study by Konrad et al. [13]. The t-test for independent samples with a power of 0.8 and an α-value of 0.05 calculated a power (1-β) of 0.95 for 84 participants. Since the sample of the study is larger than this value, we assume that the significance of the study is higher than this value.

## Results

Our study was terminated when the sample size was 90: 30 pregnant women with PE, 30 with severe PE (SPE), and 30 patients in the control group. All pregnant women who participated in the study had a singleton pregnancy, and maternal age ranged from 18 to 40 years, with a mean age of 28.77±6.37 years. The mean gestational week of the group was 35.54±3.247 weeks. The mean pulse rate of the women was 91.99±12.476 beats/min. The mean body mass index (BMI) of the study group was 29.42±4.54 kg/m^2^. Table 1 shows the comparison of demographic and disease characteristics.

**Table 1:** Baseline characteristics of the study groups. Data are expressed as median (min-max). MAP; mean arterial pressure, BMI; body mass index, AST; aspartate aminotransferase, ALT; alanine aminotransferase, LDH; lactate dehydrogenase, BUN; blood urea nitrogen, WBC; white blood cell, RDW; red cell distribution width, PLT; platelet, MPV; mean platelet volume, CRP; C-reactive protein, NLR; neutrophil/lymphocyte ratio, PLR; platelet/lymphocyte ratio

Baseline characteristics	Control group (n=30)	PE (n=30)	SPE (n=30)	p-value
Age (years)	25.5 (18-40)	27 (19-40)	31.5 (19-40)	0.020
Body mass index (kg/m^2 ^)	27 (20-36)	31 (20-35)	32 (22-38)	0.002
Gestational age (weeks)	37 (31-40)	36 (30-40)	34 (30-40)	0.018
MAP (mmHg)	76 (70-90)	104 (93-119)	120 (100-144)	<0.001
Shock index	0.88 (0.71-1.09)	0.62 (0.45-0.76)	0.61 (0.42-0.88)	<0.001
AST (U/L)	15 (9-34)	16 (10-87)	17 (8-100)	0.128
ALT (U/L)	9 (6-63)	9 (6-115)	13 (6-130)	0.045
LDH (U/L)	226 (164-296)	239 (180-791)	246 (121-791)	0.163
Uric acid (mg/dL)	4.20 (2.80-6.10)	5.85 (3.9-10.8)	6.0 (3.5-9.6)	<0.001
BUN (mg/dL)	15 (8-32)	22 (12-65)	23 (11-67)	<0.001
Creatinine (mg/dL)	0.52 (0.40-0.80)	0.60 (0.34-0.87)	0.6 (0.33-0.92)	0.094
Fibrinogen (mg/dL)	386 (230-610)	425 (186-659)	421 (204-679)	0.290
WBC(x10^3^/uL)	10.34 (5.97-17.91)	9.95 (5.58-17.66)	11.65 (6.88-18.38)	0.017
Neutrophil (x10^3^/uL)	7.57 (4.38-13.61)	7.23 (3.91-15.8)	8.5 (4.1-15.07)	0.194
Lymphocyte (x10^3^/uL)	1.91 (0.54-3.47)	1.98 (1.05-3.4)	2.33 (1.26-4.59)	0.018
RDW (x10^3^/uL)	14.05 (12.70-24.20)	14.10 (11.9-22.5)	13.65 (12.0-21.3)	0.359
PLT (x10^3^/uL)	225 (118-340)	249 (119-406)	240 (42-378)	0.618
MPV (fL)	10.3 (9.3-13.2)	11.4 (8.8-13.7)	11.2 (9.7-13.0)	0.009
CRP (mg/L)	5.0 (0.3-17.2)	5.8 (0.3-42.0)	13.5 (1.3-107.0)	0.023
NLR	3.85 (1.78-16.26)	3.82 (1.74-11.05)	3.59 (1.70-7.11)	0.780
PLR	122.49 (58.13-375.93)	132.83 (52.83-244.09)	89.67 (28.57-226.87)	0.008

The study found that the mean age was higher in the group with SPE than in the other groups (p=0.020). Gestational age was higher in women in the control group (p=0.018), shock index was higher in women in the control group (p < 0.001), and BMI value was lower in this group than in the other groups (p=0.002).

The analysis showed that the values of mean arterial pressure (MAP) increased with age, BMI, alanine aminotransferase (ALT), uric acid, blood urea nitrogen (BUN), creatinine, white blood cells (WBC), lymphocytes, mean platelet volume (MPV), and C-reactive protein (CRP), and showed moderate or weak correlation with these variables. On the other hand, the values of MAP were found to have a strong correlation with shock index value and weak and negative correlation with gestational week and platelet/lymphocyte ratio (PLR) (p < 0.05). In the study, no correlation was found between the values of MAP and the pregnancy values of aspartate aminotransferase (AST), lactate dehydrogenase (LDH), fibrinogen, neutrophils, red cell distribution width (RDW), platelets (PLT) and neutrophil/lymphocyte ratio (NLR) (p > 0.05, Table 2).

**Table 2:** Correlations of cysteinyl leukotriene values and MAP measurement of the study group according to the analysis results. MAP; mean arterial pressure, BMI; body mass index, AST; aspartate aminotransferase, ALT; alanine aminotransferase, LDH; lactate dehydrogenase, BUN; blood urea nitrogen, WBC; white blood cell, RDW; red cell distribution width, PLT; platelet, MPV; mean platelet volume, CRP; C-reactive protein, NLR; neutrophil/lymphocyte ratio, PLR; platelet/lymphocyte ratio.

Correlations of mean arterial pressure values			Correlations of cysteinyl leukotriene	
	Correlation coefficient r^s^	p-value	Correlation coefficient r^s^	p-value
Age (years)	0.237	0.025	-0.296	0.005
Gestational age (weeks)	-0.264	0.012	-0.001	0.993
MAP (mmHg)	-	-	0.030	0.777
Shock index	-0.764	<0.001	-0.039	0.717
Body mass index (kg/m^2^)	0.374	<0.001	-0.059	0.583
AST (U/L)	0.164	0.122	0.159	0.134
ALT (U/L)	0.228	0.031	-0.030	0.779
LDH (U/L)	0.189	0.074	0.136	0.202
Uric acid (mg/dL)	0.567	<0.001	-0.036	0.735
BUN (mg/dL)	0.519	<0.001	0.103	0.335
Creatinine (mg/dL)	0.244	0.020	0.017	0.874
Fibrinogen (mg/dL)	0.201	0.057	0.118	0.266
WBC(x10^3^/uL)	0.248	0.019	0.217	0.040
Neutrophil (x10^3^/uL)	0.135	0.204	0.203	0.054
Lymphocyte (x10^3^/uL)	0.300	0.004	0.069	0.517
RDW (x10^3^/uL)	-0.146	0.169	0.142	0.183
PLT (x10^3^/uL)	0.085	0.428	-0.124	0.245
MPV (fL)	0.258	0.014	0.226	0.032
CRP (mg/L)	0.277	0.008	-0.108	0.311
NLR	-0.087	0.414	0.058	0.586
PLR	-0.218	0.039	-0.087	0.415

On the other hand, leukotriene levels were found to have a weak negative correlation with age (p < 0.05), while a weak positive correlation was observed with WBC and MPV levels. CysLT level did not correlate with gestational week, MAP, shock index, BMI, AST, ALT, LDH, uric acid, BUN, creatinine, fibrinogen, neutrophil, lymphocyte, RDW, PLT, CRP, NLR, and PLR levels (p > 0.05).

**Figure 1:**
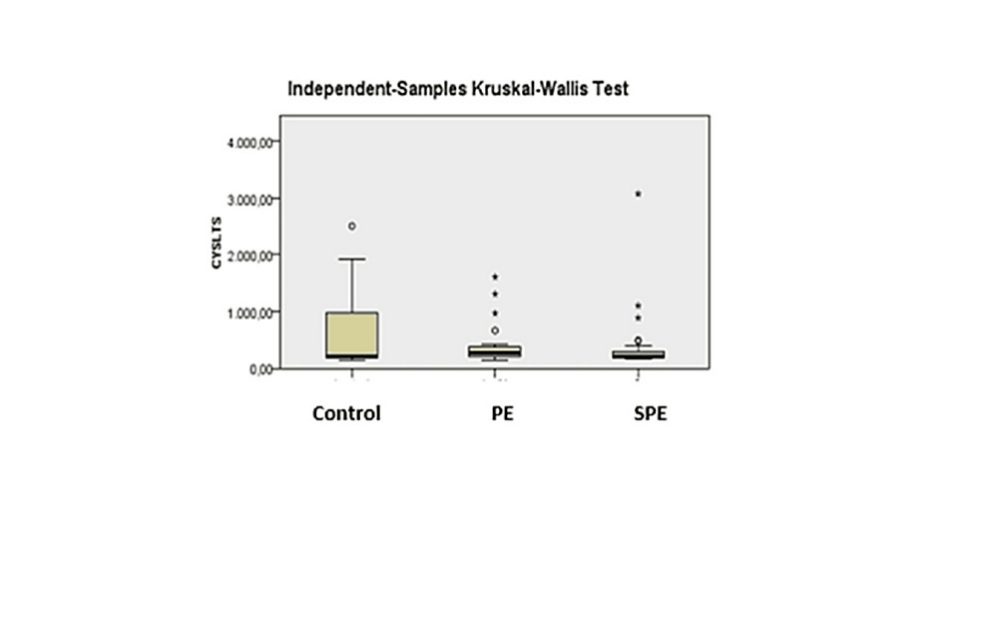
Comparison of cysteinyl leukotriene values.

The mean CysLT levels of 206.15 pg/mL for the control group, 273.2 pg/mL for PE, and 211.85 pg/mL for SPE were calculated. However, no statistically significant difference was found between the groups (p=0.707, Figure 1).

**Figure 2:**
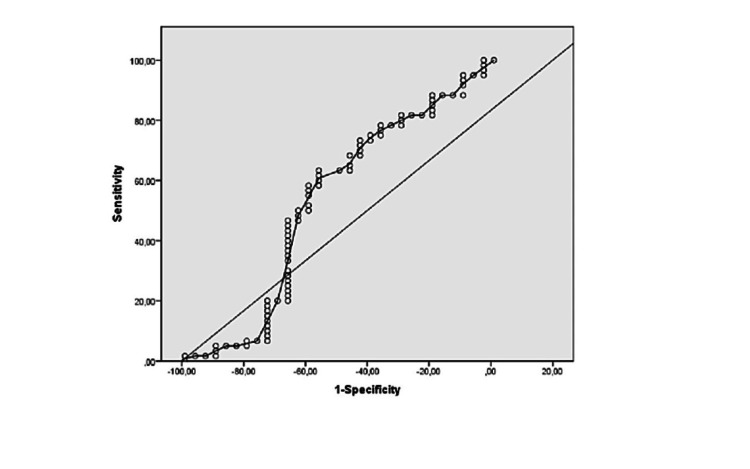
ROC curve for cysteinyl leukotriene in preeclampsia. ROC, receiver operating characteristic

The diagnostic power of ROC analysis for CysLT test in women with PE was not significant (p=0.67) (AUC under the curve [AUC] = 0.530; 95% CI = 0.422-0.636). In the analysis of ROC, the cut-off value > 966.2 was found to have optimal sensitivity (6.67%) and specificity (73.3%) (Figure 2).

 In our analysis, the cut-off value with the highest specificity and sensitivity was set at 206.4. However, because there was no difference between groups in cysteinyl leukotriene values, it is not reasonable to use a cut-off value.

## Discussion

The aim of this study was to investigate CysLT levels in PE, an inflammatory disease. It was found that CysLT levels were higher in the PE group than in the control group, but this difference was not statistically significant. There is no study in the literature on CysLT levels in preeclampsia.

To evaluate our results, the literature was reviewed in detail. It was found that many studies that examined inflammatory markers reached confounding results. It is well known that markers such as NLR, PLR, and RDW, which can be elevated by simple blood tests, indicate systemic inflammation [14]. Moreover, studies have shown that NLR, PLR, RDW, and MPV levels are elevated in patients with PE [15]. In addition, these markers have been found to be elevated in cardiovascular disease due to vascular inflammation and in some malignancies [16]. RDW has been associated with the severity and presence of hypertension in pregnant and non-pregnant patients [17,18]. On the other hand, one study reported that no change in systemic inflammatory markers was observed in women with PE compared with normal pregnant women [19]. The reason for these confusing reports is the stage of pregnancy at which the studied markers were examined. For example, it is known that in the first trimester, placentation and implantation lead to changes in maternal peripheral blood levels and especially in inflammatory markers [20]. In the study by Mannaerts et al. [13], MPV levels were found to be elevated in the first trimester of pregnancy in patients who would develop PE. Tzur and Sheiner [19] observed increased platelet levels in the first trimester in patients with PE. This is due to hypoxia occurring in the placenta, which increases erythropoietin secretion and bone marrow activity [21]. Studies have shown that platelets are activated before PE becomes clinically apparent, and platelet activation has been defined as a diagnostic tool [22,23].

Depending on the pathophysiology of PE, serum levels IL-6, CRP, and neutrophils are high, and platelet levels are low [24]. CRP is an inflammatory marker that causes cell proliferation, lipid accumulation, and thrombosis through its mediator effect. It also increases the activation of the complement system and the production of tissue factors, leading to thromboembolic events [25]. Therefore, the increase in CRP level affects many organs and impairs their functions. In our study, CRP levels were higher in the group with SPE. Similar to our study, Engin-Üstün et al. [5] reported that CRP levels above 5 mg/L pose a serious risk of morbidity and mortality for patients with PE, with a 60-fold increase in risk compared with the control group. Vickers et al. [26] have also recently shown that women with a history of PE have elevated CRP levels. In addition, maternal CRP levels have been shown to be higher in healthy pregnant women than in nonpregnant women [27]. Despite all these findings, the potential use of CRP as an early marker for PE remains controversial. Savvidou et al. [28] reported that serum CRP levels were similar in pregnant women who developed PE at 23-25 weeks of gestation and in pregnant women without complications.

From the literature review on shock index, pregnancy causes an increase in shock index [29]. In addition, high indices have been associated with postpartum hemorrhage [30]. In our study, we found that the shock index was lower in the PE group; however, we think that this is related to the increased BMI. In this regard, there is a need for BMI-adjusted studies.

There are many screening mediators used in PE severity and prediction. However, it is important that these mediators are rapid, cheap, and accessible. This is the first review that examined the CysLT score for predicting PE. The major limitation of this study is the small sample size. In addition, it is difficult to reach a general opinion because the results from a single center are compared. However, unlike the CysLT value, the influence of many factors was investigated, and an important contribution to the literature was made. In addition, the study has shed light on the literature on CysLT values in future PE studies.

## Conclusions

As a result, the shock index was low and BMI was high in the PE group. ALT, WBC, lymphocytes, CRP, PLR, and shock index were positively correlated with the value of MAP. There was no statistical difference between the CysLT levels of women in the control group, the mild PE group, and the SPE group. We believe that evaluation of our study in larger study groups in repeated studies and in different trimesters will reveal the role of CysLTs in determining the severity of PE.
